# Deciduo—Permanent Teeth

**Published:** 1891-04

**Authors:** 


### Deciduo-Permanent Teeth.

We are often brought face to face with a case in which one or more of the temporary teeth are retained in place long after they should have been shed. This is least frequent with the central incisor, occurs oftener with the first temporary molar, second temporary molar, lateral incisor and cuspid, in the order named. When any of these teeth are found in the mouth, even if partially loosened, unless there are good reasons to expect that the permanent tooth will succeed upon the extraction of the temporary, it is better to suffer it to remain. They are sometimes retained through life, and are often seen in persons at thirty or forty, though it is true that many are lost between twenty and thirty.

Not long since we saw an instance in which a dentist extracted two temporary cuspids in a young lady of eighteen; but as within two years the permanent teeth did not erupt artificial substitutes had to be made. Fill and save these teeth, and by judicious grinding away of the antagonists relieve them of a part of the burden of mastication.—*Dental Review.*

